# Commercial sex and risk of HIV, syphilis, and herpes simplex virus-2 among men who have sex with men in six Chinese cities

**DOI:** 10.1186/s12879-016-2130-x

**Published:** 2016-12-21

**Authors:** Cunge Zheng, Jun-jie Xu, Qing-hai Hu, Yan-qiu Yu, Zhen-xing Chu, Jing Zhang, Xiao-xu Han, Lin Lu, Zhe Wang, Ji-hua Fu, Xi Chen, Hong-jing Yan, Yong-jun Jiang, Wen-qing Geng, Sten H. Vermund, Han-Zhu Qian, Hong Shang

**Affiliations:** 1 Vanderbilt Institute for Global Health, Vanderbilt University Medical Center, Nashville, TN USA; 2 Department of Laboratory Medicine, Key Laboratory of AIDS Immunology of National Health and Family Planning Commission, The First Affiliated Hospital, China Medical University, 155 Nanjing North Street, Heping District, Shenyang, 110001 China; 3 Collaborative Innovation Center for Diagnosis and Treatment of Infectious Diseases, Hangzhou, China; 4 Yunnan Provincial Centers for Disease Control and Prevention, Kunming, Yunnan China; 5 Henan Provincial Centers for Disease Control and Prevention, Zhengzhou, Henan China; 6 Shandong Provincial Centers for Disease Control and Prevention, Ji’nan, Shandong China; 7 Hunan Provincial Centers for Disease Control and Prevention, Changsha, Hunan China; 8 Jiangsu Provincial Centers for Disease Control and Prevention, Nanjing, Jiangsu China; 9 Departments of Pediatrics, Vanderbilt University Medical Center, Nashville, TN USA; 10 Division of Epidemiology, Departments of Medicine, Vanderbilt University Medical Center, Nashville, TN USA

**Keywords:** Men who have sex with men, Commercial sex, HIV, Sexually transmitted infection China

## Abstract

**Background:**

Men who have sex with men (MSM) are at high risk of HIV and sexually transmitted infections (STIs) in China and globally. Engaging in commercial sex put them at even greater risk. This study estimated the prevalence of HIV/STIs among three subgroups of MSM: MSM who sold sex (MSM-selling), MSM who bought sex (MSM-buying), and non-commercial MSM (NC-MSM) and evaluated the relationship between commercial sex and HIV/STIs.

**Methods:**

We conducted a cross-sectional survey among MSM in six Chinese cities (Shenyang, Ji’nan, Changsha, Zhengzhou, Nanjing, and Kunming) from 2012 to 2013. Data on socio-demographics and sexual behaviors were collected. Serological tests were conducted to detect HIV, syphilis, and human simplex virus type 2 (HSV-2).

**Results:**

Of 3717 MSM, 6.8% were engaged in commercial sex. The overall prevalence of HIV, syphilis and HSV-2 infections was 11.1, 8.8 and 12.1%, respectively. MSM-selling had higher prevalence of HIV (13.4%), syphilis (12.1%) and HSV-2 (17.9%) than NC-MSM (10.9, 8.7 and 11.9% for HIV, syphilis and HSV-2, respectively), though the differences are not statistically significant. Among MSM-selling, HIV prevalence was significantly higher for those who found sex partners via Internet than those did not (19.4% vs. 8.1%, *P* = 0.04). Compared to NC-MSM, MSM-selling were more likely to use recreation drugs (59.3% vs. 26.3%), have unprotected anal intercourse (77.9% vs. 61.7%), and have ≥10 male sex partners (46.2% vs. 6.2%) in the past 6 months (each *P* < 0.05).

**Conclusions:**

All three subgroups of MSM in six large Chinese cities have high prevalence of HIV/STIs. Those who sell sex only have a particularly high risk of acquiring and transmitting disease, and therefore, they should be considered as a priority group in HIV/STIs surveillance and intervention programs.

**Electronic supplementary material:**

The online version of this article (doi:10.1186/s12879-016-2130-x) contains supplementary material, which is available to authorized users.

## Background

Men who have sex with men (MSM) are at a high risk of HIV infection around the world [1]. The pooled data from seven low- and middle-income Asian countries including China showed that MSM were 18.7 times more likely to have HIV infection than other reproductive-age men [2]. The risk factors associated with HIV infection among MSM include both biological and behavioral factors such as unprotected anal intercourse (UAI), substance abuse, and co-infection with other sexually transmitted infections (STIs) like syphilis and herpes simplex virus type 2 (HSV-2) [1, 3–6]. Co-infection of HIV and other STIs are prevalent among Chinese MSM [7]. Co-infection of ulcerative STIs such as syphilis and HSV-2 is of particular concern, as these STIs can increase the likelihood of HIV transmission.

Many Chinese MSM engaged in both homosexual and heterosexual activities, and some engaged in the commercial sex [8]. MSM who sell sex to men for money or goods (MSM-selling), have been found to be at higher risks of HIV and other STIs due to multiple sexual partners and high frequency of UAI [9, 10]. One study in Chengdu in southwest China showed that MSM-selling were 6.4 times more likely to have HIV infection than other MSM [11]. However, data are inconsistent. For example, a study in Shenzhen in south China found lower HIV prevalence among MSM-selling (4.6%) than other MSM (7.0%; *P* = 0.03) [12].

Along with the inconsistent data on HIV risk among MSM-selling, there are few studies among Chinese MSM who buy sex (MSM-buying). The risk of HIV/STIs has never been compared among MSM-selling, MSM-buying, and MSM who did not engage in commercial sex (NC-MSM). Such a comparison could enrich the understanding of China’s HIV epidemic and provide evidence for public health worker and policy makers to develop specific HIV intervention strategies. The aim of this study was to estimate the prevalence of HIV, syphilis and HSV-2 infections among MSM-selling, MSM-buying and NC-MSM, and assess the relationship between commercial sex engagement (either selling or buying sex) and HIV/STIs among Chinese MSM.

## Methods

### Study participants

From June 2012 to June 2013, a cross-sectional study was conducted among MSM who were living in six large Chinese cities: Shenyang, Ji’nan, Changsha, Zhengzhou, Nanjing and Kunming. These large cities represent different geographical locations, social and economic development, and HIV epidemics across China. MSM participants were recruited using multiple approaches, including advertising on gay websites and online chat rooms, outreach to gay-gathering venues (e.g., gay bars, parks, public bathhouses), and peer referrals. Subjects were eligible if they were male, 18 years or older, able to provide informed consent, and self-reported ever having sex with other men. The sample size of recruited MSM in each city was estimated based on a two-sided Z test, by using an alpha(α) of 0.05, a statistical analysis power of 90%, a HIV prevalence of 10%, and an estimate rate difference(d_0_) of 0.04, a sample size of 641 MSM in each city was needed.

### Questionnaire interview

Each participant completed a face-to-face interview with trained interviewers to collect the following information: (1) socio-demographics, including age, marital status, residency, ethnicity, education, and occupation etc.; (2) HIV/AIDS knowledge; (3) sexual and illicit drug use behaviors, including sexual orientation, sexual debut, number of sexual partners in the past 6 months, UAI, venues for seeking male sexual partners, and recreational drug use, etc.; and (4) experiences of buying or selling sex with other male partners in the past 6 months (Additional file 1). To minimize the interviewer bias, a uniform study protocol was used, and all the interviewers were trained for conducting the survey.

### Laboratory testing

A blood sample was collected from each participant for testing antibodies to HIV, syphilis and HSV-2. HIV-1 antibody was detected by enzyme-linked immunosorbent assay [ELISA] (Vironostika® HIV-1Microelisa System; bioMerieux, Durham, NC, USA), and positive tests were confirmed by Western blot test (HIV Blot 2.2 WBTM, Genelabs Diagnostics, Singapore). Rapid plasma reagin (RPR) test (RPR, Shanghai Kehua Biotechnology Ltd, Shanghai, China) was used to screen syphilis and positive tests were confirmed by the *Treponema pallidum* particle assay (TPPA) test (TPPA, Hainan Huamei Biomedicine Co, LTD, Haikou, China). Subjects with both RPR and TPPA positive testing results were considered currently infected syphilis. HSV-2 infection was determined using ELISA (HerpeSelect-2®, Focus Diagnostics, Cypress, CA, USA).

### Statistical analysis

Student t-tests were used to calculate means ± standard deviations (SD) for continuous variables and Pearson’s Chi-square test or Fisher’s exact test were used for comparing proportions (%) for categorical variables. Specifically, we compared demographic and behavioral characteristics and HIV/STI prevalence among three groups of MSM (MSM-selling, MSM-buying and NC-MSM). Univariate logistic regression analyses were performed to screen factors associated with HIV, syphilis and HSV-2 infections, respectively, and those factors which were significant at *P* < 0.05 and suggested as possible risk factors in the literature were included in multivariate logistic regression models with HIV, syphilis and HSV-2 infection as a dependent variable, separately. As the number of participants with missing data in any key variables was <10% of the total sample size, and the potential bias due to list-wise deletion in the multivariable analysis was unlikely to be significant, we did not impute the missing values using methods like the multiple imputation by chained equations. A significant level was defined as *P* < 0.05 for two-sided testing. All statistical analyses were performed using Statistical Analysis System software (SAS 9.2 for Windows; SAS Institute Inc., NC, USA).

### Ethic, consent and permissions

The study protocol and informed consent form were reviewed and approved by the institutional review board of the First Affiliated Hospital of China Medical University (2011). Study details were explained clearly for each participant and written informed consent was obtained prior to the survey.

## Results

### Demographic characteristics

**Fig. 1:**
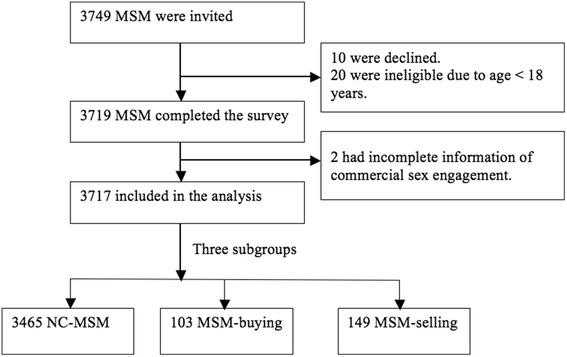
Flowchart of participant selection

A total of 3749 MSM were invited for this study, of whom 10 declined (0.3%) and 20 were ineligible due to age <18 years. Of 3719 eligible MSM participants, 2 did not answer the question about commercial sex. Therefore, 3717 participants (99.9%) were included in the analyses, with 100% of participants having HIV and syphilis outcomes and 91.2% (*n* = 3389) having HSV-2 testing results (Fig. 1).

About 6.8% of MSM participants reported being ever engaged in commercial sex in past 6 months, including 149 MSM-selling (4.0%) and 103 MSM-buying (2.8%). The median age of all participants was 26 years (IQR: 23–33 years), and the majority were single (76%), of Han ethnics (93%) and had lived in the study cities for over 6 months. About 57% had received college education or above and 14.8% were students (Table 1).

**Table 1:** Demographics and HIV, syphilis and HSV-2 prevalence by commercial sex engagement among 3717 MSM in six Chinese cities

Characteristic	Total	NC-MSM	MSM-buying	MSM-selling	*P*-value*
	*N* (%)	*N* (%)	*N* (%)	*N* (%)	
Overall	3717 (100.0)	3465 (93.2)	103 (2.8)	149 (4.0)	_
HIV positive	411 (11.1)	378 (10.9)	13 (12.6)	20 (13.4)	0.554
Syphilis positive	327 (8.8)	302 (8.7)	7 (6.8)	18 (12.1)	0.285
HSV-2 positive	409 (12.1)	380 (11.9)	12 (14.1)	17 (17.5)	0.202
Age (median, IQR, in years)	26 (23–33)	26 (23–33)	28 (24–34)	23 (21–28)	<0.001
Han majority	3447 (92.7)	3237 (93.4)	87 (84.5)	123 (82.6)	<0.001
Married with females	893 (24.0)	846 (24.4)	33 (32.0)	14(9.4)	<0.001
Living in the study city ≤6 mo.	261 (8.6)	239 (8.2)	5 (7.6)	17 (29.3)	<0.001
Student, in school	548 (14.8)	530 (15.3)	8 (7.8)	10 (6.8)	<0.001
Education: College or above	2128 (57.3)	2048 (59.1)	52 (50.5)	28 (18.8)	<0.001
Seeking male sexual partners via internet	2560 (69.0)	2439 (70.5)	59 (57.3)	62 (41.9)	<0.001
Seeking male partners locally or in other cities					
Locally	2461 (72.3)	2377 (73.8)	43 (52.4)	41 (41.0)	<0.001
In other cities	516 (15.2)	452 (14.0)	25 (30.5)	39 (39.0)	
Neither	427 (12.5)	393 (12.2)	14 (17.1)	20 (20.0)	
Number of MSM partners in past 6 months					
0–2	2315 (63.2)	2250 (65.5)	37 (39.0)	28 (21.5)	<0.001
3–9	1052 (28.7)	973 (28.3)	37 (39.0)	42 (32.3)	
≥10	294 (8.0)	213 (6.2)	21 (22.1)	60 (46.2)	
Use of any recreational drugs in past 6 months	886 (27.5)	804 (26.3)	31 (40.3)	51 (59.3)	<0.001
UAI in past 6 months	2320 (62.4)	2136 (61.7)	68 (66.0)	116 (77.9)	<0.001
Predominant role in anal sex					
Equal	1228 (33.9)	1136 (33.4)	31 (31.6)	61 (49.2)	0.001
Insertive	1180 (32.5)	1122 (33.0)	36 (36.7)	22 (17.4)	
Receptive	1219 (33.6)	1147 (33.7)	31 (31.6)	41 (33.1)	
Having sex with female, in past 6 months	675 (18.2)	613 (17.7)	34 (22.8)	28 (27.2)	0.016

*HSV-2* human herpes virus type 2, *UAI* Unprotected anal intercourse, *IQR* interquartile range

*Chi-square test

Overall, MSM-selling were younger, less likely to be married and more likely to living in the study cities for only 6 months or less, and had lower education level than both MSM-buying and NC-MSM. Fewer student MSM were found in the subgroups of MSM-buying or MSM-selling than NC-MSM (each *P* < 0.001).

### Prevalence of HIV, syphilis and HSV-2

The overall prevalence of HIV, syphilis and HSV-2 infection was 11.1, 8.8 and 12.1%, respectively (Table 1). MSM-selling had higher HIV prevalence than NC-MSM and MSM-buying (13.4% vs. 10.9% vs. 12.6%, *p* = 0.55). MSM-selling also had higher prevalence of syphilis and HSV-2 (12.1 and 17.5%, respectively) than the other the two groups though the difference was not statistically significant (*P* > 0.05).

**Fig. 2:**
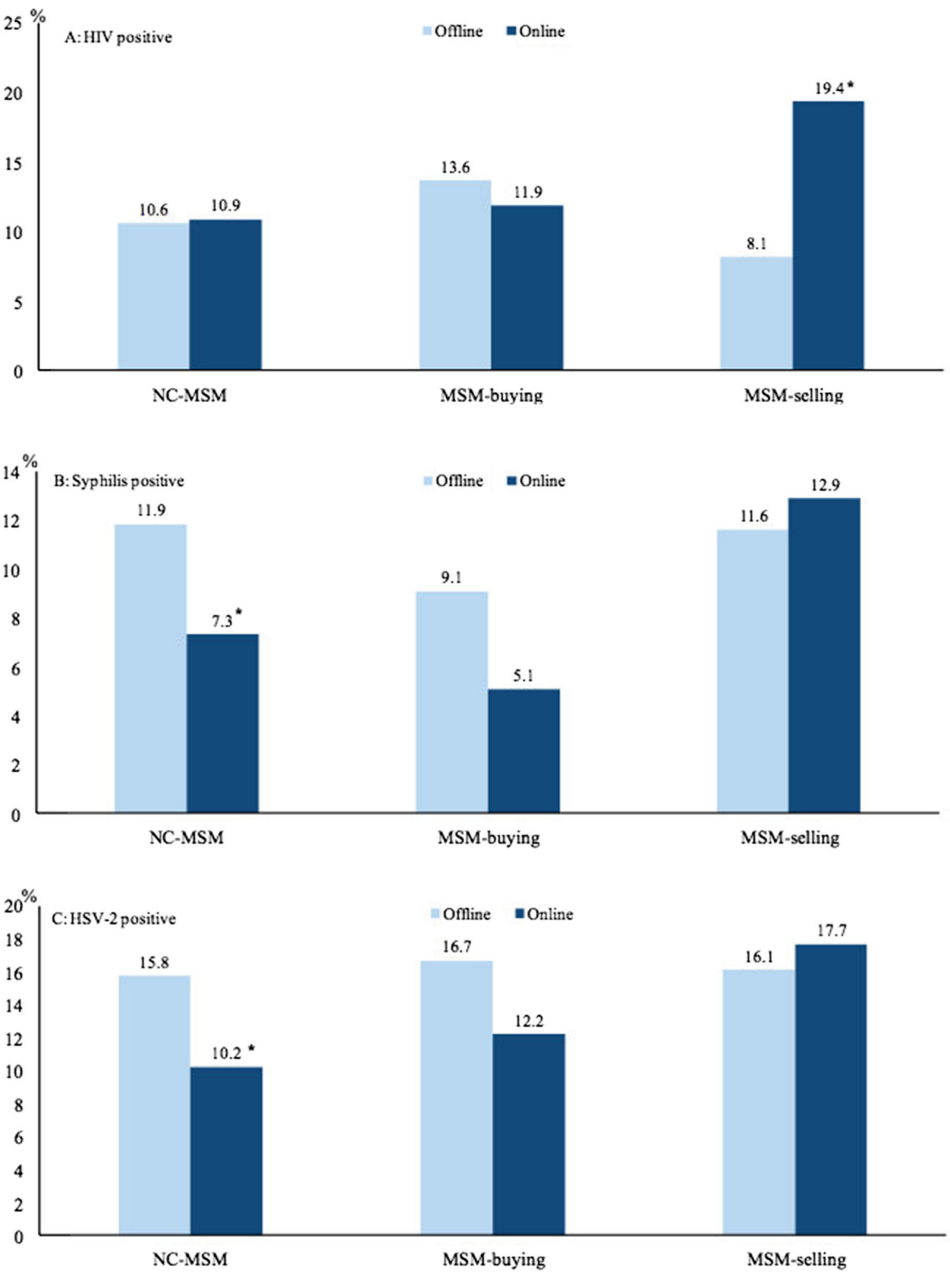
The prevalence of HIV (**a**), syphilis (**b**), and HSV-2 (**c**) among MSM seeking male sex partners online by commercial sex engagement (HSV-2 = human herpes virus type 2; *chi-square test, *p* < 0.05)

When the prevalence of HIV was broken down by seeking male sex partners online, among MSM-selling the prevalence of HIV infection was significantly higher for those seeking sex partners online than those seeking male sex partners offline 19.4% (95% CI, 10.4–31.4%) vs. 8.1% (95% CI, 3.3–16.1%), *P* = 0.04, Fig. 2a). Among NC-MSM, those who seek male partners online had significantly lower prevalence of syphilis/HSV-2 infection than NC-MSM seeking male sex partners offline with syphilis prevalence of 7.3% (95% CI, 6.3–8.5%) vs. 11.9% (95% CI, 9.9–14.0%, *P* < 0.01, Fig. 2b) and HSV-2 prevalence of 10.2% (95% CI, 9.0–11.6%) vs. 15.8% (95% CI, 13.4–18.3%, *P* < 0.01, Fig. 2c).

### Sexual behavior of participants

Almost 70% of participants reported seeking male sexual partners online, 72% had sought male partners in local cities and 18.2% had sex with females in the past 6 months (Table 1). Over 62% were engaged in UAI, 27.5% used recreational drugs and 36.8% had 3 or more male sex partners in past 6 months.

Fewer MSM-selling reported seeking male sex partners online than NC-MSM (41.9% vs. 70.5%). More MSM-selling had ≥10 male sex partners, used recreational drugs, were engaged in UAI, and had sex with a female in the past 6 months.

### Factors associated with HIV, syphilis and HSV-2 infection

The unadjusted odds of having HIV was 1.2 (95% CI, 0.7–2.1) and 1.3 (95% CI, 0.8–2.1) for MSM-buying and MSM-selling, in comparison to NC-MSM (Table 2). Engagement in commercial sex was not independently associated with HIV/STIs after adjusted for socio-demographics, co-infections and risk behaviors. In the multivariable model, the prevalence of HIV was significantly associated with several socio-demographic factors (shorter length of residency, non-student occupation, and lower educational level), sexual behaviors (multiple sexual partners, use of drugs, UAI and predominantly reception anal sex), and biological co-factors (co-infection with syphilis or HSV-2) (Table 2).

**Table 2:** Factors associated with HIV infection among 3717 Chinese MSM

Characteristic	No. of MSM HIV positive (%)	Crude OR (95% CI)	*P*-value	AOR (95% CI)	*P*-value
Commercial sex					
NC-MSM	378 (10.9)	Ref.	0.55	Ref.	0.88
MSM-buying	13 (12.6)	1.2 (0.7–2.1)		1.1 (0.5–2.5)	
MSM-selling	20 (13.4)	1.3 (0.8–2.1)		1.2 (0.5–2.6)	
Syphilis positive					
No	333 (9.8)	Ref.	<0.001	Ref.	<0.001
Yes	78 (23.9)	2.9 (2.2–3.8)		2.0 (1.4–2.8)	
HSV-2 positive					
No	275 (9.2)	Ref.	<0.001	Ref.	<0.001
Yes	88 (21.5)	2.7 (2.1–3.5)		2.3 (1.7–3.1)	
Age (per year)		1.0 (1.0–1.1)	0.001	1.0 (0.9–1.0)	0.24
Married to a woman					
No	293 (10.4)	Ref.	0.018	Ref.	0.18
Yes	118 (13.2)	1.3 (1.0–1.7)		1.2 (0.9–1.6)	
Time living in the study city					
>6 months	317 (11.4)	Ref.	0.023	Ref.	0.031
≤6 months	42 (16.1)	1.5 (1.1–2.1)		1.5 (1.0–2.2)	
Student					
No	376 (11.9)	Ref.	<0.001	Ref.	0.002
Yes	35 (6.4)	0.5 (0.4–0.7)		0.5 (0.3–0.8)	
Education					
College or above	210 (9.9)	Ref.	0.008	Ref.	0.15
High school or below	201 (12.6)	1.3 (1.1–1.6)		1.2 (0.9–1.5)	
Seeking partners online					
No	121 (10.5)	Ref.	0.6	Ref.	0.72
Yes	284 (11.1)	1.1 (0.8–1.3)		1.1 (0.8–1.4)	
Number of male sex partners in past 6 months					
0–2	220 (9.5)	Ref.	<0.001	Ref.	0.023
3–9	147 (14.0)	1.5 (1.2–1.9)		1.4 (1.1–1.8)	
≥10	34 (11.6)	1.2 (0.8–1.8)		1.1 (0.7–1.7)	
Drug use in past 6 months					
No	237 (10.1)	Ref.	<0.001	Ref.	<0.001
Yes	144 (16.3)	1.7 (1.4–2.1)		1.7 (1.3–2.2)	
UAI in past 6 months					
No	123 (8.8)	Ref.	<0.001	Ref.	0.001
Yes	288 (12.4)	1.5 (1.2–1.8)		1.5 (1.2–1.9)	
Predominant anal sex position					
Receptive	265 (12.8)	Ref.	<0.001	Ref.	0.014
Insertive	139 (9.0)	0.7 (0.5–0.8)		0.7 (0.6–0.9)	

The risks of current syphilis infection were similar to those of HIV infection, with a few differences (Table 3). In multivariable analysis, syphilis was associated with HIV and/or HSV-2 infections, older age, more sex partners, drug use, and UAI. The length of city residency and predominant receptive anal sex position were not significant for syphilis infection. For HSV-2 infection, co-infections with HIV and/or syphilis, non-students, lower educational status, and not seeking partners online were significantly associated (Table 4).

**Table 3:** Factors associated with syphilis infection among 3717 Chinese MSM

Characteristic	No. of MSM syphilis positive (%)	Crude OR (95% CI)	*P*-value	AOR (95% CI)	*P*-value
Commercial sex					
NC-MSM	302 (8.7)	Ref.	0.28	Ref.	0.262
MSM-buying	7 (6.8)	0.8 (0.4–1.7)		0.5 (0.2–1.4)	
MSM-selling	18 (12.1)	1.4 (0.9–2.4)		1.5 (0.7–3.3)	
HIV positive					
No	249 (7.5)	Ref.	<0.001	Ref.	<0.001
Yes	78 (19.0)	2.9 (2.2–3.8)		2.0 (1.5–2.8)	
HSV-2 positive					
No	208 (7.0)	Ref.	<0.001	Ref.	<0.001
Yes	81 (19.8)	3.3 (2.5–4.4)		2.7 (2.0–3.7)	
Age (per year)		1.0 (1.0–1.1)	0.001	1.0 (1.0–1.1)	0.002
Married to a woman					
No	222 (7.9)	Ref.	<0.001	Ref.	0.45
Yes	105 (11.8)	1.6 (1.2–2.0)		0.9 (0.6–1.2)	
Time living in the study city					
>6 months	244 (8.7)	Ref.	0.31	_	_
≤6 months	18 (6.9)	0.8 (0.5–1.3)			
Student					
No	305 (9.6)	Ref.	<0.001	Ref.	0.170
Yes	22 (4)	0.4 (0.3–0.6)		0.7 (0.4–1.2)	
Education					
College or above	145 (6.8)	Ref.	<0.001	Ref.	0.080
High school or below	182 (11.5)	1.8 (1.4–2.2)		1.3 (1.0–1.7)	
Seeking partners online					
No	135 (11.7)	Ref.	<0.001	Ref.	0.082
Yes	190 (7.4)	0.6 (0.5–0.8)		0.8 (0.6–1.0)	
Number of male sex partners in past 6 months					
0–2	175 (7.6)	Ref.	0.002	Ref.	0.002
3–9	113 (10.7)	1.5 (1.1–1.9)		1.5 (1.1–1.9)	
≥10	35 (11.9)	1.7 (1.1–2.4)		1.5 (1.0–2.5)	
Drug use in past 6 months					
No	182 (7.8)	Ref.	0.024	Ref.	0.007
Yes	91 (10.3)	1.4 (1.0–1.8)		1.5 (1.1–2.0)	
UAI in past 6 months					
No	102 (7.3)	Ref.	0.013	Ref.	0.038
Yes	225 (9.7)	1.4 (1.1–1.7)		1.3 (1.0–1.8)	
Predominant position in anal sex					
Receptive	265 (12.8)	Ref.	0.23	_	_
Insertive	139 (9.0)	0.9 (0.7–1.1)

**Table 4:** Factors associated with HSV-2 infection among 3389 Chinese MSM

Characteristic	No. of MSM HSV-2 positive (%)	Crude OR (95% CI)	*P*-value	AOR (95% CI)	*P*-value
Commercial sex					
NC-MSM	380 (11.8)	Ref.	0.20	Ref.	0.52
MSM-buying	12 (14.1)	1.2 (0.7–2.3)		1.5 (0.7–2.9)	
MSM-selling	17 (17.5)	1.6 (0.9–2.7)		1.2 (0.5–2.6)	
HIV positive					
No	88 (24.2)	Ref.	<0.001	Ref.	<0.001
Yes	321 (10.6)	2.7 (2.1–3.5)		2.3 ((1.8–3.1)	
Syphilis positive					
No	81 (28)	Ref.	<0.001	Ref.	<0.001
Yes	328 (10.6)	3.3 (2.5–4.4)		2.7 (2.0–3.7)	
Age (per year)		1.1 (1.0–1.1)	<0.001	1.0 (1.0–1.1)	<0.001
Married to a woman					
No	266 (10.4)	Ref.	<0.001	Ref.	0.61
Yes	143 (17)	1.8 (1.4–2.2)		0.9 (0.7–1.2)	
Time living in the study city					
>6 months	333 (11.9)	Ref.	0.88	_	_
≤6 months	32 (12.3)	1.0 (0.7–1.5)			
Student					
No	391 (13.6)	Ref.	<0.001	Ref.	<0.001
Yes	18 (3.5)	0.2 (0.1–0.4)		0.4 (0.2–0.7)	
Education					
College or above	194 (9.8)	Ref.	<0.001	Ref.	0.37
High school or below	215 (15.3)	1.7 (1.4–2.1)		1.1 (0.9–1.4)	
Seeking partners online					
No	159 (15.8)	Ref.	<0.001	Ref.	0.28
Yes	247 (10.4)	0.6 (0.5–0.8)		0.9 (0.7–1.1)	
Number of male sex partners in past 6 months					
0–2	236 (11.1)	Ref.	0.12	Ref.	0.27
3–9	129 (13.3)	1.2 (1.0–1.5)		1.2 (0.9–1.5)	
≥10	36 (14.2)	1.3 (0.9–1.9)		1.3 (0.8–1.9)	
Drug use in past 6 months					
No	278 (11.9)	Ref.	0.88	Ref.	0.64
Yes	93 (11.7)	1.0 (0.8–1.3)		1.1 (0.8–1.4)	
UAI in past 6 months					
No	154 (11.6)	Ref.	0.54	Ref.	0.19
Yes	255 (12.3)	1.1 (0.9–1.3)		0.9 (0.7–1.1)	
Predominant anal sex position					
Receptive	134 (11.6)	Ref.	0.35	_	_
Insertive	127 (11.6)	0.9 (0.7–1.1)			

## Discussion

Our study showed that HIV, syphilis and HSV-2 infections were prevalent in all of three subpopulations of MSM (MSM-selling, MSM-buying and NC-MSM) in China. However, among MSM-selling, HIV prevalence was significantly higher for those seeking sex partners online than those seeking sex partners offline. To our knowledge, these findings were scarcely reported before. Additionally, MSM-selling reported to have risk sexual behaviors more frequently than other two groups.

In this cross-sectional study, the rates of HIV (11.1%) and HSV-2 (12.1%) among MSM overall were comparable to another study in multiple cities [13], which reported 10% of HIV infection and 16% of HSV-2 infection among MSM. But the overall HIV prevalence was more than 2-fold higher than that in a national survey across 61 cities during 2008–2009 (4.9%), and the overall syphilis rate (8.8%) was lower than that of national survey (12%) [14]. The prevalence of HIV/STIs varied by geographical distributions across China [14, 15], which was in agreement with our findings in these six cities. To investigate the association between the prevalence of STIs and commercial sex activities, the advantages of using pooled data from multiple sites include is to while having heterogeneity associated with regions.

Compared to MSM without commercial sex, the prevalence of HIV/STIs is usually higher among MSM sex workers [11, 16, 17]. Our findings also demonstrated this trend that MSM with commercial sex had higher HIV/STIs prevalence than those without commercial sex, as suggested in other study [18], though the prevalence of HIV, syphilis and HSV-2 was not significant different among MSM-selling, MSM-buying and NC-MSM. One noticeable characteristic of MSM-selling in the current study was that they were significantly younger than MSM-buying or NC-MSM. Hence, shorter HIV exposure period may decrease their cumulative HIV acquiring risk.

It is usually assumed that female commercial sex workers play a bridging role for HIV heterosexual transmission [19]. Our study results enriched the understanding between commercial sexual activity and HIV transmission risk. We revealed that MSM-selling may contribute to both hetero- and homo- sexual transmission, which has special public health significance in China. We found that a larger number of MSM-selling had sex with females in past 6 months and the prevalence of HIV/STIs was also significantly higher among married MSM. Additionally, more MSM-selling and MSM-buying reported looking for male sex partners in other cities than NC-MSM. Given the facts that almost one-third of MSM-selling lived in study cities for ≤6 months and that short local residency was significantly related to HIV infection, domestic migration and commercial sex activities in other cities might potentially facilitate the transmission of HIV among MSM across geographical locations [20]. These findings indicated the potential bridges of HIV infection across sexes, from MSM to general female population, and then possibly to children [21].

HIV prevalence among MSM was significantly associated with co-infections with syphilis and/or HSV-2, as expected [13, 14, 22]. Genital ulcers can facilitate HIV transmission by breaching protective mucosal barriers and recruiting susceptible immune cells (e.g., CD4 T-helper cells, macrophages) to the site of infection [23], and can also create portals of entry for HIV to access susceptible cells. Another potential explanation is that HIV shares the same sexual risk factors with these STIs.

We found that MSM-selling were significantly different from MSM-buying and NC-MSM regarding socio-demographics and sexual behaviors. As well as being younger, MSM-selling were less educated, more likely to be unmarried, and highly migratory. Compared to NC-MSM, more MSM-selling had ≥10 male sex partners (46%), had sex with females (27%), used recreational drugs (59%) and had UAI (78%) in past 6 months. The profiles of characteristics among MSM-selling in this study were similar to findings in other studies on MSM sex workers [20, 24]. We can prudently suggest that the prevalence of HIV/STIs among MSM-selling in our study may rise higher than NC-MSM and MSM-buying (median ages 23 vs. 26 vs. 28 years old, respectively) after given it 3 or 5 more years of HIV exposure period, since they also had highest prevalence of syphilis and HSV-2 which were significantly associated with HIV as mentioned before.

Usually MSM seeking sexual partners via internet were involved in comparatively higher HIV risk behavior such as recreational drug use, multiple partners and unprotected anal sex [25]. A study which reviewed HIV surveillance data from 33 U.S. states between 1999 and 2008 claimed that a major online personals ad site, *craigslist*®, accounted for a 16% increase in HIV infection [26]. The studies from China had different results. In a prospective cohort study in China, the HIV incidence was similar between MSM who sought male sex partners online and those offline (8.6% vs 7.4%, *p* = 0.6) [27]. A meta-analysis that investigated the relationship between the prevalence of HIV/syphilis and the types of venues seeking male sex partners found that men seeking sex via sauna were 2.27 and 1.61 times more likely to have HIV and syphilis infection than men seeking partners online, respectively [14]. Wu et al. [21] suggested that MSM seeking male sex partners online are relatively younger, better-educated, less engagement in UAI, less involvement in commercial sex, and have more HIV knowledge. These characteristics might explain the lower prevalence of HIV/syphilis among MSM seeking male sex partners online in China.

Among all MSM participants, we found that seeking male sex partners online was not significantly related to HIV infection, but significantly related to syphilis and HSV-2 infection as a protective factor, even after adjusted for other variables. These findings were in agreement with previous studies [14, 27]. However, these results must be interpreted with some cautions. We further compared the prevalence of HIV/STI infection between MSM seeking male partners online and those offline for the three MSM subpopulations separately. Among MSM-selling, we observed that MSM seeking male sex partners online had significantly higher HIV prevalence than those seeking sex offline. The significantly lower prevalence of syphilis and HSV-2 among MSM seeking male partners online was only observed among NC-MSM, not among MSM-buying or MSM-selling. These results echoed our finding that MSM-selling is a significantly different subgroup from NC-MSM and MSM-buying. MSM-selling had higher prevalence of unprotected anal intercourse and recreational drug use. As suggested by Zhao et al., sexual risk behaviors among MSM may be influenced by venue-specific characteristics [28]. MSM-selling might use different websites to seek male sex partners and have different perception of online seeking, which might increase their HIV risk or facilitate HIV/STIs transmission. We lacked the statistical power to investigate the interaction effect of seeking male sex partners online and commercial sex activities on HIV/STI infections.

The strengths of our study include large sample size, diverse geography, and multiple recruitment venues. This study also has limitations. First, behavioral data were collected via interviewer-administered questionnaire survey, which might lead to underreporting of commercial sex due to stigma. We took several measures to minimize this information bias, including providing standardized trainings for all interviewers, not collecting any personal identifiable information from participants, and explaining to participants about the study purpose and anonymity nature prior to conducting the interviews. Second, the number of MSM with commercial sex experience was moderate, which limited the statistical power for MSM subgroup comparisons. Third, while the refusal rate in our study was low, we cannot assess the proportion of MSM who heard about the study but did not come for eligibility screening. Last, since our study sample represents MSM in urban China only, we cannot generalize findings to rural or small township residents. We speculate that MSM in rural or small town venues may have more clandestine social networks with stronger local stigma against homosexuality, with attendant differences in sexual behaviors and HIV/STI prevalence. As our study is the first study comparing the risk of HIV/STIs among Chinese MSM with different commercial sex activities, the findings provide valuable information for HIV/STI prevention interventions among these high-risk subgroups.

## Conclusions

The high prevalence of risky sexual behaviors among MSM-selling may not only promote HIV acquiring and transmission risk in this specific subpopulation, but also in other MSM subpopulations and general population. Moreover, the subgroup of MSM-selling who sought male sex partners online had higher HIV prevalence. The increasing use of internet in China may influence MSM preferences in venues seeking male sex partners. All MSM in China, especially those MSM-selling subgroups seeking sex partners online, should be considered as a key population, to be given highest priority in disease surveillance and intervention programs.

## Additional file

Additional file 1: Questionnair and Related Questions_Commercial Sex and Sexual behavior Survey among MSM in Big Cities of China. (PDF 82 kb)

## Abbreviations

ELISA: Enzyme-linked immunosorbent assay
HIV: Human immunodeficiency virus
HSV-2: Herpes simplex virus type 2
MSM: Men who have sex with men
MSM-buying: MSM who buy sex
MSM-selling: MSM who sell sex to men for money or goods
NC-MSM: MSM who did not engage in commercial sex
RPR: Rapid plasma reagin
SAS: Statistical Analysis System
SD: Standard deviations
STIs: Transmitted infections
TPPA: *Treponema pallidum* particle assay
UAI: Unprotected anal intercourse
