# Supplementary material for: Commercial sex and risk of HIV, syphilis, and herpes simplex virus-2 among men who have sex with men in six Chinese cities
Source: BMC Infect Dis. 2016 Dec 21;16:765. doi: 10.1186/s12879-016-2130-x (PMC5178086; doi:10.1186/s12879-016-2130-x)
Supplement: Additional file 1: — Questionnair and Related Questions_Commercial Sex and Sexual behavior Survey among MSM in Big Cities of China. (PDF 82 kb) [file 12879_2016_2130_MOESM1_ESM.pdf]

File Name: Commercial Sex and Sexual behavior Survey among MSM in Big Cities of China

Title of Data: Demographic Information

Description:

1. PID(Sig digital number):_________
2. Date of birth (YYYY/MM/DD):_______________
3. Permanent residence address: ______province______city_____street/town____village/buiding
4. Marriage status:
   a. Never married; b. Married with a heterosexual woman; c. Married with a homosexual woman; d. Divorce or widower; e. Live together with a female;
   f. Live together with a male; g. others_______________
5. Ethnicity:
   a. Han; b. Others (please specify__________)
6. Education:
   a. Less than elementary school; b. Elementary school; c. Middle school;
   d. High school or technique school; e. College or above
7. Are you currently a student now?
   a. Yes; b. No
8. How long have you been lived in the current city?
   a. <3 months; b. 3-6 months; c. 7-12 months; d. >1 year, ≤2 years; e. >2 years

Title of Data: Sexual Behavior and recreational drug using behavior

Description:

1. In the recent 6 months, did you have sex with a man by offering money, drug or other goods to him?

   a. Yes; b. No

2. In the recent 6 months, did you have sex with a man who paid you money, drug or other goods?

   a. Yes; b. No

3. How old were you when you had first insertion sex? ___________________(in years)

4. The gender of your first sexual partner:

   a. Male; b. Female (please move to question 4);

5. How old were you when you had first sex with a man? ___________________(in years)

6. What is your predominant role in anal sex?

   a. Insertive; b. Receptive; c. Equal

7. In the recent 6 months, did you look for male sex partners in your current residential city?

   a. Yes (the number of male sex partners _____); b. No

8. In the recent 6 months, did you look for male sex partners in other cities?

   a. Yes (the number of male sex partners _____); b. No

9. In the recent 6 months, how often do you use condom during the anal sex?

   a. Never; b. Some times; c. Every time

10. In the recent 6 months, how many different male partners did you have sex with?

    a. _____________(number of partners); b. Unclear

11. In the recent 6 months, did you had sex with female? 1) Yes; 2) No

12. Do you regularly seek male sex partners in the following sites? (Please state the percentage of frequencies, e.g. Internet (90%); Park (10%). The total should be added up to 100 %.)

    a. Internet ----------------------1) Yes (______%); 2) No

    b. Hotel -------- -----------------1) Yes (______%); 2) No

    c. Bar------------------------- ---- 1) Yes (______%); 2) No

d. Night club ---------------- ---- 1) Yes (______%);   2) No

e. Public bathhouse------- ---- 1) Yes (______%);   2) No

f. Public restroom--------- ---- 1) Yes (______%);   2) No

g. Park----------------------------- 1) Yes (______%);   2) No

h. MSM club--------------------- 1) Yes (______%);   2) No

i. Others--------------------- ---- 1) Yes (______%);   2) No

13. In the recent 6 months, did you buy (by using money or gifts) sexual service from casual male partners?   1) Yes;   2) No

14. How many above male sexual partners did you have in the recent 6 months?_______

15. In the recent 6 months, did you provide sexual service to any casual male partners to get his/their money or gifts?   1) Yes;   2) No

16. How many above male sexual partners did you have in the recent 6 months?_______

17. In the recent 6 months, did you use the below recreation drugs?

a.Popper[Alky Nitrites]   1) Yes; 2) No

b. Ecstasy   1) Yes; 2) No

c. Ice   1) Yes; 2) No

d. Amphetamine   1) Yes; 2) No

e. Tramadol   1) Yes; 2) No

f. Ketamine   1) Yes; 2) No

g. Others_________   1) Yes; 2) No

Name of the questionnair investigator：_________   Survey date：(YYYY/MM/DD):______

Name of the questionnair reviewer: _________   Review date：(YYYY/MM/DD):______
